# COVID-19 symptom duration: evidence from a survey of infected workers in Tokyo

**DOI:** 10.3389/fpubh.2026.1808868

**Published:** 2026-06-25

**Authors:** Asako Chiba, Taisuke Nakata, Naohisa Shobako, Reo Takaku

**Affiliations:** 1 Department of Economics, Keio University, Minato-ku, Tokyo, Japan; 2 Graduate School of Economics, The University of Tokyo, Bunkyo-ku, Tokyo, Japan; 3 Graduate School of Agriculture, Kyoto University, Sakyo-ku, Kyoto, Japan; 4 Graduate School of Economics, Hitotsubashi University, Kunitachi, Tokyo, Japan

**Keywords:** attributes, COVID-19, duration (time), prevalence, symptoms

## Abstract

**Introduction:**

The prevalence and duration of symptoms following COVID-19 infection vary substantially across individuals. However, little is known about the relationship between workers' job characteristics and the persistence of post-COVID symptoms. This study investigated the prevalence and duration of symptoms following COVID-19 infection and examined their associations with occupational characteristics.

**Methods:**

We conducted a large-scale retrospective survey of infected workers in February 2023. The survey collected information on symptom duration, job characteristics, and individual demographic, health, and socio-economic characteristics. We estimated adjusted odds ratios for prolonged symptoms using a three-month threshold while controlling for potential confounding factors. A sensitivity analysis was conducted using a one-month symptom-duration threshold.

**Results:**

Most infected workers experienced symptoms for less than one month. However, approximately 5% reported symptoms lasting three months or longer. Part-time workers had higher adjusted odds of experiencing symptoms for three months or more. Service workers, managers, and transportation workers tended to have lower odds of prolonged symptoms. However, the associations observed for occupational groups were not robust in the sensitivity analysis using a one-month threshold.

**Discussion:**

The findings suggest that the persistence of symptoms following COVID-19 infection varies across employment types and occupations. In particular, part-time workers may be at greater risk of long-lasting symptoms.

## Introduction

The COVID-19 pandemic caused countless human tragedies. According to the World Health Organization, more than 7 million deaths have been reported worldwide as of October 2025 [WHO (1)]. This number is likely to be a significant understatement of the actual death figure due to underreporting. Even when COVID-19 did not kill a person, it often negatively impacted the infected person's life via severe and sometimes long-lasting symptoms.

A substantial body of evidence has documented the prevalence and persistence of COVID-19 symptoms—a condition commonly referred to as long COVID or post-COVID condition—across diverse populations (2–12). The WHO defines post-COVID condition as symptoms that occur in individuals with a history of COVID-19 infection, usually 3 months from onset, lasting at least 2 months, and that cannot be explained by an alternative diagnosis (13–15). Studies have identified a range of individual-level factors associated with higher risk of prolonged symptoms, including female sex (16–19), pre-existing health conditions (20–23), obesity (24, 25), and unvaccinated status (26–28).

In Japan, several studies have examined the prevalence and characteristics of long COVID, including those by Hirahata et al. (29), Kutsuna et al. (30), Sugiyama et al. (31), and Asakura et al. (32). Studies focusing on hospitalized or severely ill patients tend to report a high prevalence of long-lasting symptoms (31), while community-based studies including non-hospitalized patients report considerably lower prevalence (30, 32). However, despite Japan's large working-age population and the known economic burden of COVID-19 absenteeism, little is known about the prevalence and duration of COVID-19 symptoms specifically among workers, or about how employment type and occupational characteristics relate to symptom duration. Occupational factors such as employment type, job demands, access to occupational health services, and eligibility for paid sick leave may plausibly influence both the severity of acute infection and the recovery trajectory, yet these factors have been understudied in the existing literature on long COVID.

With this gap, the objectives of this study are to (1) document the prevalence and duration of COVID-19 symptoms among workers in Tokyo, and (2) identify individual and occupational characteristics associated with self-reported prolonged symptoms (lasting approximately 3 months or longer).

## Materials and methods

We conducted our retrospective survey from 07/02/2023 to 16/02/2023, in collaboration with Cross Marketing Inc., an online market research company based in Japan. We first accessed the anonymized data for research purposes on 17/02/2023. In the survey, we restricted our attention to those who lived in Tokyo and were aged between 20 and 64 at the time of our survey. This age range was chosen because our survey targets workers, and individuals between the ages of 20 and 64 represent the working-age population in Japan. Our target was workers who had gotten infected with COVID-19 at least once during the COVID-19 crisis. We conducted a screening survey to identify those who had a job in March 2020—right before the COVID-19 crisis intensified—and had gotten infected with COVID-19 at least once by the time of the survey. For those who had been infected multiple times, we asked about their most recent infection. The distributions of gender and age were matched to those in the Population Census. We received responses from 10,000 individuals.

Our survey was approved by the Ethics Review Committee of the University of Tokyo (Approval No.22-382). Prior to participation, we informed respondents that the survey was anonymous and that their responses would be used for research purposes. Only those who agreed proceeded to the questionnaire.

**Figure 1:**
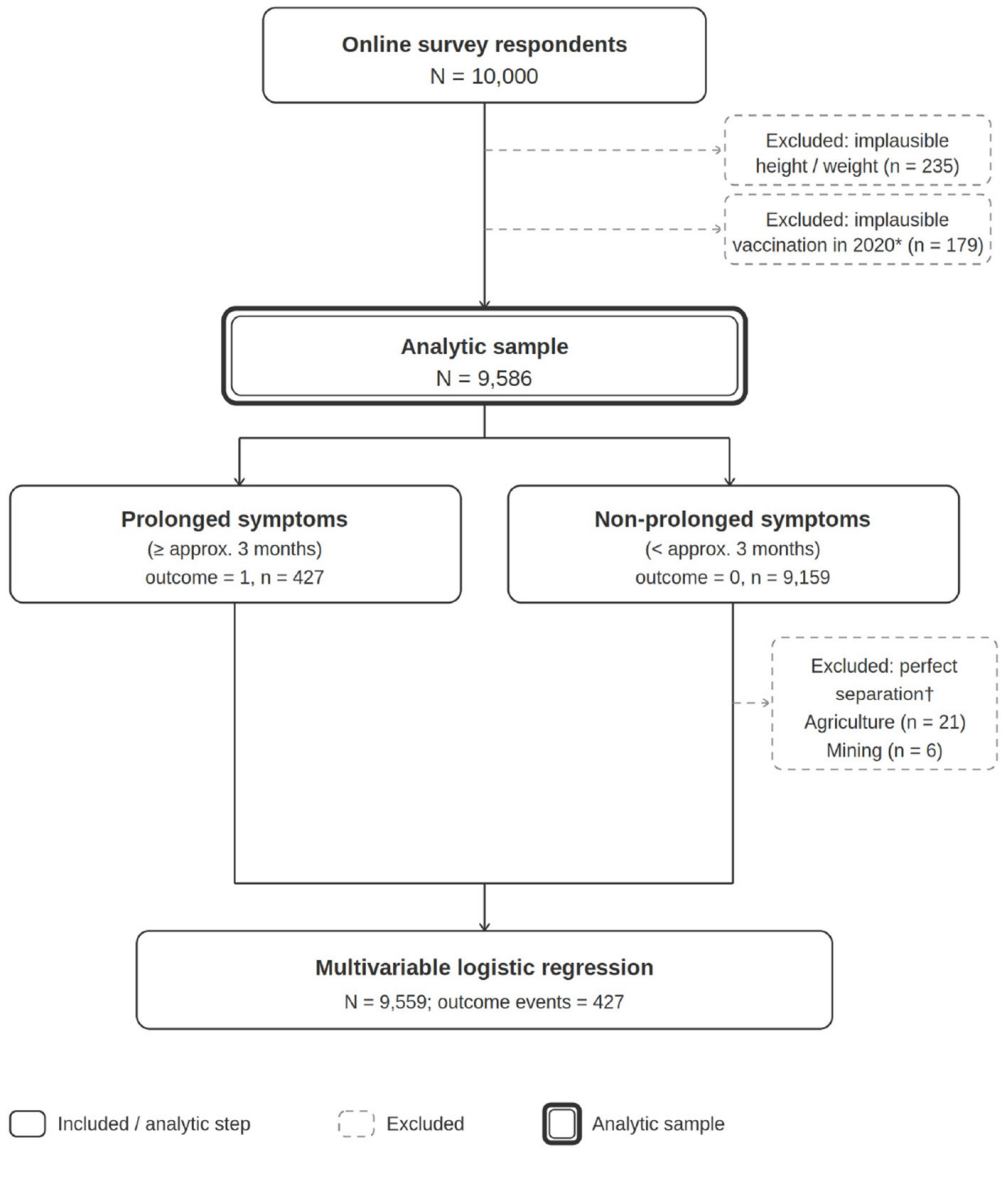
Participant flow diagram. *Vaccines were not available in Japan in 2020. Respondents reporting vaccination at the time of a 2020 infection were excluded as their responses likely reflect recall error. ^†^Agriculture (*n* = 21) and Mining (*n* = 6) were omitted from the logistic regression because no outcome events (prolonged symptoms) were observed in these occupations (perfect separation).

We excluded 235 participants with implausible anthropometric values (height below 100 cm or above 250 cm, or weight below 30 kg or above 200 kg), as respondents reporting such extreme values were considered to have not answered the survey seriously. One hundred and seventy-nine participants were further excluded because they reported being vaccinated at the time of their 2020 infection; as COVID-19 vaccines were not yet available in Japan in 2020, these responses likely reflect recall error. The final analytic sample comprised 9,586 participants. In the multivariable logistic regression, 27 additional participants were excluded due to perfect separation: no prolonged symptom cases were observed among workers in Agriculture (*n* = 21) or Mining (*n* = 6), making it impossible to estimate coefficients for these occupational categories. The effective regression sample, therefore, comprised 9,559 participants. The derivation of the analytic sample is summarized in Figure 1.

In the survey, we asked the respondents a wide range of questions. The questions can be roughly categorized into (i) their COVID-19 symptoms, (ii) individual characteristics including demographic, health, and socio-economic, and (iii) job characteristics.

For (i), we asked our respondents when they got infected with COVID-19 and asked them to select all symptoms they experienced among a list of ten symptoms: fever, sore throat, fatigue, cough, headache, joint pain, taste/smell loss, brain fog, sleep disorder, and hair loss. We also asked about the duration of each symptom. Rather than asking for an exact number of days, we provided 11 choices prefixed with “About” (e.g., “About 1–2 weeks”, “About 1–2 months”), ranging from “0 day” to “more than 6 months,” with 0 days indicating that the infected worker did not suffer from the symptom. This design was intended to reduce respondent cognitive burden and encourage more accurate recall of approximate symptom duration; we acknowledge that it does not allow precise measurement of exact symptom duration. Symptom duration was assessed separately for each of the ten symptoms; the primary outcome in the regression analysis is defined as the maximum duration across all symptoms reported by each participant.

For (ii), we asked our respondents their age, gender, education, income in 2019, whether they lived with older adults or infants at the time of infection, drinking habits, smoking habits, pre-existing health conditions, and their vaccination status at the time of their COVID-19 infection (specifically, whether they had received a booster shot, one or more vaccine doses without a booster, or no vaccine prior to infection). We also asked them the type of employment—regular or non-regular workers; self-employed, part-time, or full-time—industry, and the availability of remote work.

For (iii), we asked our respondents about their jobs following the occupational classification by the Ministry of Internal Affairs and Communications. Agriculture includes jobs related to farming, forestry, fishing, and livestock. Mining covers occupations involved in extracting natural resources such as coal, minerals, oil, and gas. Sales refers to positions that involve selling products. Service refers to providing customer-facing services. Manager positions are leadership roles responsible for planning, organizing, and directing business or organizational operations. Clerical Worker roles involve administrative and office tasks like data entry, record keeping, and scheduling. Transport and Machine Operation includes jobs where workers operate vehicles or heavy machinery to transport goods, people, or materials. Manufacturing Process refers to roles in production facilities where workers assemble products, operate machines, or inspect goods. Professional and Engineering jobs are highly skilled roles requiring specialized education or training. Security Worker jobs are focused on maintaining safety and protecting people or property. Others is a broad category that includes jobs not easily classified in the above groups.

Table 1 shows summary statistics for individual characteristics. The distribution of gender and age is close to that in Tokyo by construction. Education distribution shows a larger share of junior-college/university graduates than in official statistics: it is 79.2% in our survey, whereas it is 70.4% for workers aged between 20 and 64 in Tokyo according to the Population Census. Income distribution shows a smaller share of high-income workers than in official statistics: it is 18.0% in our survey, whereas it is 30.0% for workers in Tokyo according to the Statistical Survey of Actual Status for Salary in the Private Sector. Employment-type distribution is close to the official statistics. The shares of regular, contract, part-time, and self-employed/other workers are 60.3%, 8.6%, 17.7%, 13.4%, respectively, whereas they are 57.6%, 4.6%, 23.3%, and 14.5% according to the Employment Status Survey. These data suggest that the participants represent the residents in Tokyo reasonably well.

**Table 1:** Summary statistics.

Attributes	Obs.
Age	
20–39	3,688 (38.5%)
40–59	5,184 (54.1%)
60-	714 (7.4%)
Gender	
Male	4,768 (49.7%)
Female	4,818 (50.3%)
Cohabitants	
Infants	1,600 (16.7%)
Older adults	1,304 (13.6%)
Education	
College or more	7,596 (79.2%)
Income	
≥6 mil. yen (2022)	1,730 (18.0%)
Employment type	
Regular	5,781 (60.3%)
Contract	823 (8.6%)
Part-time	1,697 (17.7%)
Self-employed and Others	1,285 (13.4%)
Habits	
Drinking	5,289 (55.2%)
Smoking	1,839 (19.2%)
Pre-existing health conditions	
Obesity (BMI ≥ 25)	1,686 (17.6%)
Cancer	180 (1.9%)
Cerebrovascular disease	67 (0.7%)
Respiratory disease	254 (2.6%)
CVD	162 (1.7%)
Digestive disease	200 (2.1%)
ESD	237 (2.5%)
Kidney disease	67 (0.7%)
Blood system disease	121 (1.3%)
Vaccine	
Unvaccinated	1,584 (16.5%)
Took booster shot	4,777 (49.8%)
Severity	
Artificial respirator/ECMO	74 (0.8%)
*N*	9,586 (100.0%)

In the next section, we will first examine symptom duration. In the following section, we present regression analyses of factors associated with Self-reported prolonged symptoms. The primary outcome is a binary variable equal to one if an individual reports at least one symptom lasting approximately 3 months or longer, and zero otherwise. This 3-month threshold was chosen to align with the WHO definition of post-COVID condition, which describes symptoms persisting for at least 2 months with onset within 3 months of acute COVID-19 infection (13–15). Because our outcome is based on self-report rather than clinical diagnosis, we use the term “self-reported prolonged symptoms” rather than “post-COVID syndrome” or “long COVID” throughout this paper. As a sensitivity analysis, we also estimated a binary logistic regression model using a 1-month-or-more threshold as the outcome (i.e., at least one symptom lasting approximately 4 weeks or longer), presented in Appendix Figure 3.

We used a multivariable logistic regression (logit) model to examine factors associated with the primary outcome. Fourteen independent variables were entered simultaneously into the model: age, sex, cohabitation with older adults/infants, education, income, employment type, occupation, drinking habit, smoking habit, obesity, pre-existing health conditions, vaccination status, and infection period. This approach was motivated by prior evidence linking these characteristics to long-lasting COVID-19 symptoms and by our primary interest in occupational factors. The primary prespecified exposure of interest was job characteristics, motivated by prior literature on occupational health disparities; associations with other individual characteristics are considered secondary and exploratory. Acute disease severity (requiring artificial respirator or ECMO) was not included as a covariate because it may represent an intermediate variable on the causal pathway between baseline characteristics and prolonged symptoms and because the number of participants in this category was extremely small (*N* = 74, 0.8%), precluding reliable estimation. Results are presented as adjusted odds ratios (aOR) with 95% confidence intervals (CIs). Statistical significance was defined as *p* < 0.05.

## Results

###  Symptom duration

Table 2 presents the distribution of symptom durations lasting (i) less than 1 month, (ii) 1 month or longer, but less than about 3 months, and (iii) about 3 months or longer. In this table, we measure the duration for which at least one symptom lasts. About 5% of our respondents did not suffer from any symptoms. For simplicity, we include cases with no symptoms in the group for less than 1 month. The top line is for the entire sample from 2020 to 2023, whereas the following three lines are for year-by-year. We combine 2023 with 2022 because our survey covers only the first month of 2023.

**Table 2:** Distribution of symptom durations: total and year-by-year.

Year	< 1 month	1–3 months	3+ months	Total
Total	8,534 (89.0%)	625 (6.5%)	427 (4.5%)	9,586 (100.0%)
2020	169 (86.2%)	11 (5.6%)	16 (8.2%)	196 (100.0%)
2021	784 (83.2%)	64 (6.8%)	94 (10.0%)	942 (100.0%)
2022–2023	7,581 (89.7%)	550 (6.5%)	317 (3.8%)	8,448 (100.0%)

For the majority of our respondents, the symptoms lasted less than 1 month (89.0%). 6.5% of patients experienced symptoms lasting for 1 month or longer, but less than about 3 months, while 4.5% experienced symptoms lasting for about 3 months or longer.

The distribution of symptom durations is broadly similar across 2020 and 2021. The distribution is 86.2%, 5.6%, and 8.2% in 2020, whereas it is 83.2%, 6.8%, and 10.0% in 2021. The distribution in 2022–2023 differs somewhat from those in 2020 and 2021. In particular, the proportion of self-reported prolonged symptoms (3+ months) is noticeably lower in 2022-2023 than in 2020 and 2021 (3.8% vs. 8.2% and 10.0%). This lower prevalence of prolonged symptoms in 2022–2023 relative to other years means the higher prevalence of symptoms lasting less than 1 month. The prevalence of short symptoms is 89.7% in 2022–2023, whereas it is 86.2 and 83.2% in 2020 and 2021, respectively.

The distribution of symptom durations depends importantly on the nature of the symptom. According to Table 3, most of the infected workers with fever—the most common symptom in our survey—suffered from the symptom for less than 1 month (88.2%), and only 4.8% of them suffered from the symptom for about 3 months or longer. On the other hand, 58.7% of infected workers with hair loss suffered from the symptom for less than 1 month, and 23.8% of them suffered from the symptom for about 3 months or longer.

**Table 3:** Distribution of symptom durations: by symptoms.

	< 1 month	1–3 months	3+ months	Total
Fever	88.2%	7.0%	4.8%	8,182
Sore throat	88.1%	7.2%	4.8%	7,291
Fatigue	87.2%	7.5%	5.3%	7,178
Headache	85.9%	8.1%	6.0%	4,869
Joint pain	84.5%	8.6%	6.9%	3,760
Cough	84.0%	9.7%	6.3%	5,505
Taste/Smell loss	81.7%	9.9%	8.4%	2,573
Sleep disorder	73.2%	12.7%	14.1%	1,254
Less concentration	71.9%	13.8%	14.2%	1,488
Hair loss	58.7%	17.5%	23.8%	760

The more common a symptom is, the more short-lived it is likely to be. The proportion of the symptom duration being less than 1 month decreases as the occurrence of the symptom, shown in the last column of the table, becomes less common. Conversely, the proportion of symptom duration exceeding about 3 months increases as the occurrence of the symptom becomes less frequent.

###  Associated factors

**Figure 2:**
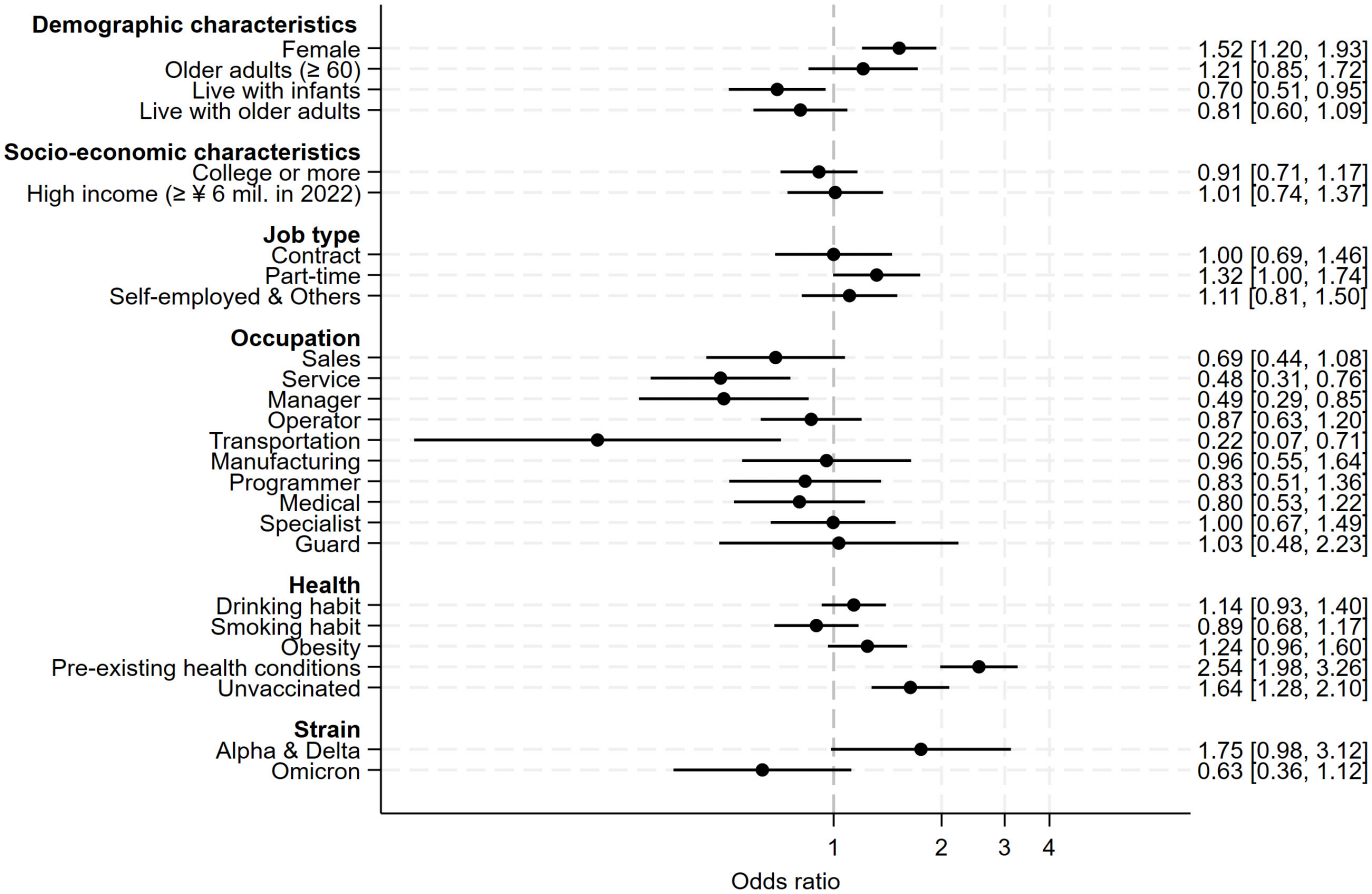
Factors associated with self-reported prolonged symptoms. Adjusted odds ratios (aOR) with 95% confidence intervals from a multivariable logistic regression model. All variables displayed are simultaneously adjusted for one another, including infection period (Wuhan as reference). Reference categories: Age 20–59; Male; No cohabitant infants or older adults; Non-college graduate; Income <6 million yen; Regular employment; Others (occupation); No drinking habit; No smoking habit; No obesity; No pre-existing health conditions; Vaccinated; Infection period 2022–2023 (Wuhan as reference for strain). *N* = 9,559, outcome events = 427.

Figure 2 shows the results of the regression analysis. For demographic characteristics, females (aOR 1.52, 95% CI 1.20–1.93) and the older adults (aOR 1.21, 95% CI 0.85–1.72) are more likely to experience self-reported prolonged symptoms than males and those below 60 years old, though the association is not statistically significant for the older adults. Those living with infants or the older adults are less likely to experience self-reported prolonged symptoms than those not living with them, though not in a statistically significant way for those living with the older adults.

For socio-economic characteristics, college graduates and high-income workers were just as likely to experience self-reported prolonged symptoms as non-college graduates and lower-income workers.

For job characteristics, contract workers were just as likely to experience self-reported prolonged symptoms as regular workers. In contrast, part-time workers were more likely to experience self-reported prolonged symptoms than regular workers (aOR 1.32, 95% CI 1.00–1.74). We also find that workers in some occupations—service workers (aOR 0.48, 95% CI 0.31–0.76), managers (aOR 0.49, 95% CI 0.29–0.85), and transportation workers (aOR 0.22, 95% CI 0.07–0.71)—were less likely to suffer from a self-reported prolonged symptom in a statistically significant way.

Finally, for health characteristics, those with drinking habits are more likely to experience self-reported prolonged symptoms, though these associations are not statistically significant. Those with smoking habits are less likely to experience self-reported prolonged symptoms, also in a statistically insignificant way. Those with pre-existing health conditions and unvaccinated workers were more likely to suffer from a self-reported prolonged symptom in a statistically significant way.

For infection period, compared to the reference category (Wuhan), those infected during the Alpha & Delta period had higher adjusted odds of self-reported prolonged symptoms, while those infected during the Omicron period had lower adjusted odds. However, neither association reached statistical significance.

The sensitivity analysis using a 1-month-or-more threshold (self-reported symptoms lasting approximately 4 weeks or longer) is presented in Appendix Figure 3. In this alternative model, the higher odds for female and the lower odds for those who live with infants are still statistically significant. Neither job type showed a statistically significant difference. Transportation workers were statistically significantly less likely to experience prolonged symptoms. Drinking and smoking habits were associated with lower odds, with statistical significance for smoking habits. Those with pre-existing health conditions and unvaccinated workers showed statistically significantly higher odds. For infection period, those infected during the Alpha & Delta period were statistically significantly more likely to experience prolonged symptoms compared to the Wuhan period, while the Omicron period did not show statistical significant difference.

These results are broadly consistent in direction with the primary 3-month threshold model. We note, however, that the association between part-time employment and prolonged symptoms was not statistically significant in the sensitivity analysis, nor were the occupational associations. The robustness of health-related findings (pre-existing conditions, vaccination status) and the infection period associations is more strongly supported across both models.

Strain-specific exploratory analyses for the Alpha & Delta and Omicron periods are presented in Appendix Figures 4, 5. The Wuhan-period subgroup was excluded from these exploratory analyses due to the very small sample size (*N* = 196, outcome events = 16), which precludes reliable estimation. We caution against drawing strong conclusions from these strain-specific estimates.

## Discussion

This study aimed to document the prevalence and duration of COVID-19 symptoms among workers in Tokyo and to identify individual and occupational characteristics associated with self-reported prolonged symptoms. The main contribution of our analysis is to document the association between COVID-19 symptom duration and work-related characteristics.

We find that part-time workers had higher adjusted odds of self-reported prolonged symptoms than regular workers in the primary analysis. We also find that workers in some occupations—service workers, managers, and transportation workers—were less likely to suffer from a self-reported prolonged symptom in a statistically significant way. Nevertheless, these associations did not reach statistical significance in a sensitivity analysis using a 1-month threshold. The higher odds among part-time workers may reflect limited access to paid sick leave and healthcare, reduced capacity to rest during acute illness, and socioeconomic vulnerability not fully captured by the income variable in our model. Occupational differences may further reflect variation in physical activity levels, psychosocial stress, and workplace exposures across job categories. Given the large number of comparisons across occupational and individual characteristics, some statistically significant findings may be due to chance; we encourage readers to place greater weight on the direction and magnitude of effect estimates and their confidence intervals rather than on binary significance tests.

In addition to work-related characteristics, we provide new evidence on correlations between COVID-19 symptom duration and other individual characteristics that have been studied before. For demographic characteristics, we find that those living with infants are less likely to experience self-reported prolonged symptoms, consistent with Wood et al. (33) and Solomon et al. (34). For socio-economic characteristics, we do not find a statistically significant relationship between COVID symptoms and income, whereas Kim (35) reports a positive relationship in the U.S. This discrepancy may reflect differences in healthcare systems and social safety nets between Japan and the United States. In Japan, universal health insurance coverage may reduce income-related disparities in access to care, potentially attenuating the association between income and symptom duration that is more pronounced in the U.S. context.

Finally, for health characteristics, we found that pre-existing health conditions were associated with self-reported prolonged symptoms. This finding is consistent with a body of evidence suggesting that underlying health vulnerabilities—such as chronic respiratory, cardiovascular, or psychiatric conditions—may impair immune regulation and recovery, thereby prolonging symptom duration (26). The biological mechanisms likely involve chronic inflammation, immune dysregulation, and reduced physiological reserve, all of which may predispose individuals to more severe or persistent post-acute sequelae following COVID-19 infection.

Unvaccinated workers were also likely to experience a prolonged symptoms. This finding is consistent with the broader literature suggesting that COVID-19 vaccination reduces not only the severity of acute illness but also the risk of persistent symptoms (27, 28). Vaccination may attenuate prolonged symptoms through several mechanisms, including reducing initial viral load, limiting the inflammatory cascade during acute infection, and promoting more efficient immune clearance. Our result reinforces the importance of vaccination as a public health measure not only for preventing acute COVID-19 but also for mitigating its longer-term sequelae.

The key strengths of our study are as follows. First, the large sample size (*N* = 9,586) provides adequate statistical power for multivariable analyses across a broad set of covariates. Second, the inclusion of detailed occupational and employment-type variables is a distinctive feature rarely found in COVID-19 symptom studies. Third, the survey was conducted in February 2023, while the majority of infections occurred in 2022, limiting the recall period for most participants.

Our analysis is subject to several limitations. First, our survey is retrospective and thus potentially suffers from recall bias. Because our survey was conducted in February 2023 and the majority of our survey participants experienced COVID-19 infections in 2022, the experience of infection was within less than 1 year for the majority of our survey participants. This consideration does mitigate the recall bias concern, but does not eliminate it. Second, our survey targeted workers who had been infected at least once and asked about their most recent infection. As a result, our analysis does not account for the possible impact of multiple infections on symptom duration, which may be an important source of bias. Third, we rely on self-reports by directly asking participants about their COVID-19 symptoms, rather than collecting clinical diagnoses. Symptoms are inherently subjective, and some participants might have had difficulty attributing specific symptoms to COVID-19 rather than other causes.

Fourth, although we adjusted for a broad set of individual and occupational characteristics, residual confounding from unmeasured variables—such as detailed lifestyle factors, access to healthcare, and specific workplace exposures—cannot be excluded, as is inherent in observational research. Fifth, the use of an online panel may introduce selection bias. The over-representation of college graduates (79.2 vs. 70.4% in official statistics) and under-representation of high-income workers (18.0 vs. 30.0%) relative to official statistics suggest that our findings may not be fully generalizable to all infected workers in Tokyo. Sixth, while our survey asked respondents about the timing of their COVID-19 infection, we did not collect information on the timing of vaccination. We only asked how many vaccine doses respondents had received at the time of infection. As a result, the relationship between vaccination timing and symptom duration—which may be an important determinant of vaccine effectiveness against long-lasting symptoms—cannot be examined in the current study and remains a direction for future research.

Finally, inclusion and exclusion criteria may limit the generalizability of our findings. Our survey was restricted to individuals aged 20–64, reflecting the working-age population in Japan; our findings may not be generalizable to younger or older individuals, who may have different symptom profiles and risk factors for prolonged COVID-19 symptoms. We also excluded 235 participants who reported anthropometric values outside plausible ranges (height below 100 cm or above 250 cm, or weight below 30 kg or above 200 kg). While we cannot rule out that some of these participants genuinely had such extreme values, the very small number excluded (2.3% of the initial sample) suggests that the impact on our estimates is negligible. In addition, 27 participants in Agriculture (*n* = 21) and Mining (*n* = 6) occupations were excluded from the multivariable logistic regression due to perfect separation—no prolonged symptom cases were observed in these groups, making it impossible to estimate the corresponding coefficients. While these exclusions are a consequence of the small sample sizes of these occupational categories, they imply that our regression results do not cover the full set of industries. Given these limitations, caution is warranted when interpreting our results.

## Conclusion

We conducted a large-scale retrospective survey of infected workers in February 2023 in Tokyo to better understand COVID-19 symptoms. While most respondents experienced acute symptoms lasting less than 1 month, a substantial fraction experienced symptoms for more than 1 month, and some for more than 3 months. We analyzed the association between individual characteristics and the prevalence of self-reported prolonged symptoms lasting more than 3 months.

Several individual characteristics are associated with self-reported prolonged symptoms. Older workers tend to be more vulnerable, while those living with the older adults tend to be less so. Socio-economic characteristics such as education and income were not meaningfully associated with prolonged symptoms. Health-related factors—particularly pre-existing health conditions and unvaccinated status—were associated with higher odds for the self-reported prolonged symptoms. Among job types, part-time workers showed higher odds of prolonged symptoms in the primary analysis. Some occupational categories also showed tendencies toward lower odds. Nevertheless, the association with job types and occupational categories did not reach statistical significance consistently across analyses.

These findings have implications for labor and public health policy. The higher odds of self-reported prolonged symptoms among part-time workers suggest that this group—who typically have limited access to paid sick leave and occupational health services in Japan—may be particularly vulnerable during a pandemic. Policymakers may consider strengthening occupational health support for non-regular workers as part of future pandemic preparedness planning. Additionally, our results suggest that employment type and occupation should be systematically collected in COVID-19 surveillance systems to enable monitoring of work-related disparities in post-COVID outcomes.

## Data Availability

The raw data supporting the conclusions of this article will be made available by the authors, without undue reservation.
