# Supplementary material for: COVID-19 symptom duration: evidence from a survey of infected workers in Tokyo
Source: Front Public Health. 2026 Jun 25;14:1808868. doi: 10.3389/fpubh.2026.1808868 (PMC13346185; doi:10.3389/fpubh.2026.1808868)
Supplement: Supplementary file 1 [file Supplementary_file_1.pdf]

Appendix

Sensitivity analysis: alternative outcome definition

Fig 3 presents the results of the sensitivity analysis using a one-month-or-more outcome definition (self-reported symptoms lasting approximately four weeks or longer). In this model, transportation workers were statistically significantly less likely to experience prolonged symptoms; those with pre-existing health conditions and unvaccinated workers were statistically significantly more likely; and those infected during the Alpha & Delta period had statistically significantly higher odds compared to the Wuhan period. The direction of associations is broadly consistent with the primary three-month threshold model.

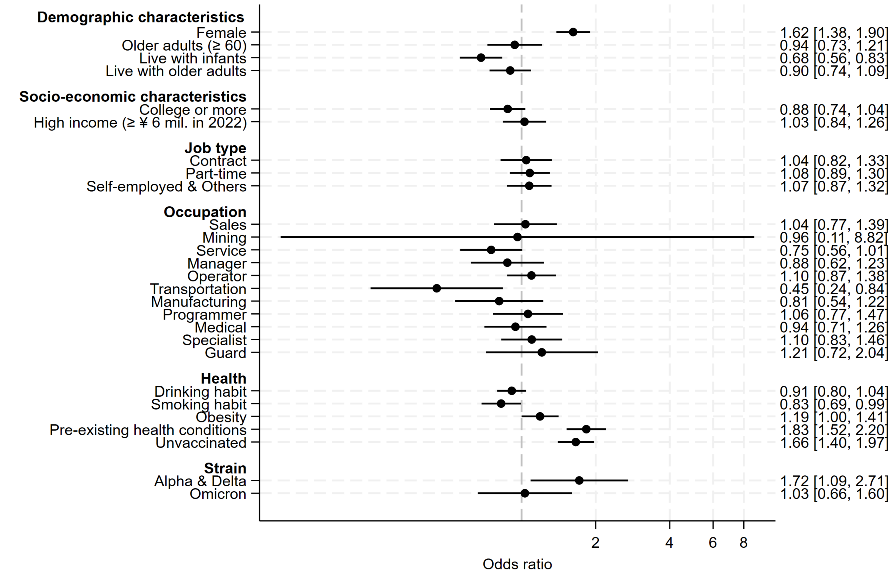

Figure 3. Factors associated with self-reported symptoms lasting approximately one month or longer

Note: Adjusted odds ratios (aOR) with 95% confidence intervals from a multivariable logistic regression model using an alternative binary outcome (at least one symptom lasting approximately four weeks or longer). All variables simultaneously adjusted, including infection period (Wuhan as reference). Same reference categories as Fig 2. N = 9,565, outcome events = 1,052.

Strain-specific exploratory analyses

Figs 4 and 5 present exploratory strain-specific regression analyses for the Alpha & Delta and Omicron periods, respectively. The Wuhan-period subgroup (N = 196, outcome events = 16) was excluded due to the very small sample size, which precludes reliable estimation. These estimates should be interpreted with caution and no strong conclusions should be drawn from individual strain-specific results.

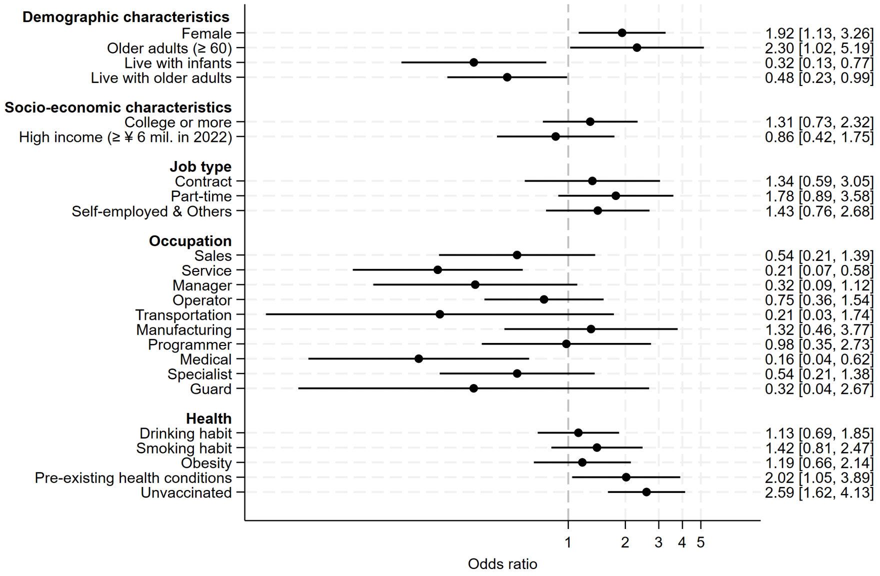

Figure 4. Factors associated with self-reported prolonged symptoms, Alpha & Delta period

Note: Exploratory subgroup analysis. aOR from multivariable logistic regression; same reference categories as Fig 2. Estimates should be interpreted with caution. N = 931, outcome events = 94.

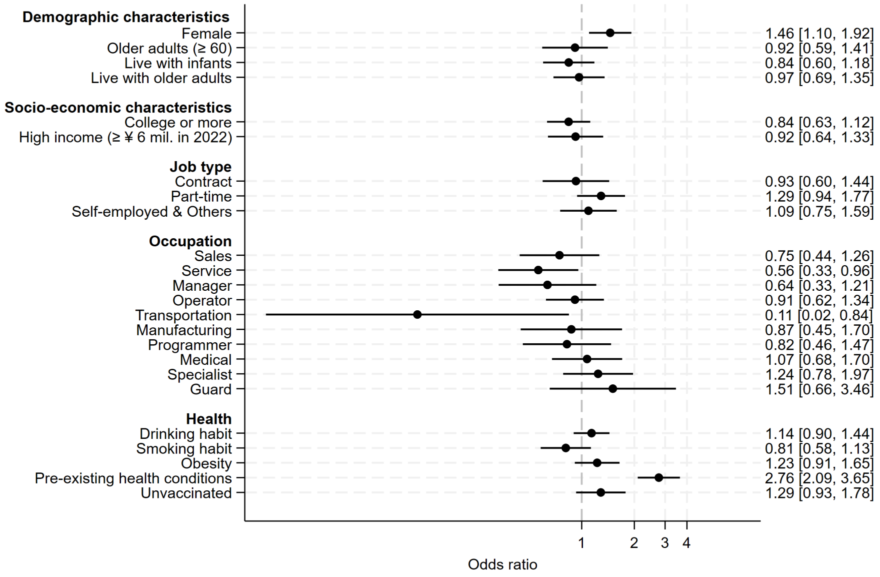

Figure 5. Factors associated with self-reported prolonged symptoms, Omicron period

Note: Exploratory subgroup analysis. aOR from multivariable logistic regression; same reference categories as Fig 2. Estimates should be interpreted with caution. N = 8,433, outcome events = 317.
